# Tissue pretreatment for LC–MS/MS analysis of PUFA and eicosanoid distribution in mouse brain and liver

**DOI:** 10.1007/s00216-019-02170-w

**Published:** 2019-12-21

**Authors:** Madlen Reinicke, Juliane Dorow, Karoline Bischof, Judith Leyh, Ingo Bechmann, Uta Ceglarek

**Affiliations:** 1 Institute of Laboratory Medicine, Clinical Chemistry and Molecular Diagnostics, Leipzig University, Liebigstr. 27, 04103 Leipzig, Germany; 2 Institute of Anatomy, Leipzig University, Liebigstr. 13, 04103 Leipzig, Germany; 3 LIFE – Leipzig Research Center for Civilization Diseases, Leipzig University, Philipp-Rosenthal-Str. 27, 04103 Leipzig, Germany

**Keywords:** Extraction, Eicosanoids, Brain lipids, Mass spectrometry, Polyunsaturated fatty acids, Tissue sample preparation

## Abstract

Polyunsaturated fatty acids (PUFAs) and eicosanoids are important mediators of inflammation. The functional role of eicosanoids in metabolic-syndrome-related diseases has been extensively studied. However, their role in neuroinflammation and the development of neurodegenerative diseases is still unclear. The aim of this study was the development of a sample pretreatment protocol for the simultaneous analysis of PUFAs and eicosanoids in mouse liver and brain. Liver and brain samples of male wild-type C57BL/6J mice (11–122 mg) were used to investigate conditions for tissue rinsing, homogenization, extraction, and storage. A targeted liquid chromatography–negative electrospray ionization tandem mass spectrometry method was applied to quantify 7 PUFAs and 94 eicosanoids. The final pretreatment protocol consisted of a 5-min homogenization step by sonication in 650 μL *n*-hexane/2-propanol (60:40 v/v) containing 2,6-di-*tert*-butyl-4-methylphenol at 50 μg/mL. Homogenates representing 1 mg tissue were extracted in a single step with *n*-hexane/2-propanol (60:40 v/v) containing 0.1% formic acid. Autoxidation was prevented by addition of 2,6-di-*tert*-butyl-4-methylphenol at 50 μg/mL and keeping the samples at 4 °C during sample preparation. Extracts were dried under nitrogen and reconstituted in liquid chromatography eluent before analysis. Recovery was determined to range from 45% to 149% for both liver and brain tissue. Within-run and between-run variability ranged between 7% and 18% for PUFAs and between 1% and 24% for eicosanoids. In liver, 7 PUFAs and 15 eicosanoids were quantified; in brain, 6 PUFAs and 21 eicosanoids had significant differences within the brain substructures. In conclusion, a robust and reproducible sample preparation protocol for the multiplexed analysis of PUFAs and eicosanoids by liquid chromatography–tandem mass spectrometry in liver and discrete brain substructures was developed.

**Electronic supplementary material:**

The online version of this article (10.1007/s00216-019-02170-w) contains supplementary material, which is available to authorized users.

## Introduction

Eicosanoids are potent lipid mediators of inflammation, synthesized from polyunsaturated fatty acids (PUFAs) via cyclooxygenase (COX), lipoxygenases, and cytochrome P450. Low-grade inflammation is crucial for the formation of obesity-linked insulin resistance and fatty liver disease, leading to peripheral metabolic disease [1, 2], which can also affect the central nervous system [3, 4]. PUFAs and eicosanoids play an important role in development and synaptogenesis [5, 6], regulation of temperature and sleep [7], synaptic activity, plasticity, and memory acquisition [8, 9], and protection of the brain against glutamate toxicity and excitotoxic injury [10, 11]. A deficiency in PUFAs can lead to retarded visual acuity, cognitive impairment, or cerebellar dysfunction [12, 13]. There is some evidence that a disrupted eicosanoid metabolism plays a role in neurodegenerative diseases [14]. However, a thorough understanding of eicosanoid mechanisms in liver and brain requires a suitable analytical method. Eicosanoid analysis is challenging because of the very low concentrations (picograms per milliliter) in blood, urine, and tissues, in vitro metabolism, and autoxidation. For the extraction of PUFAs and eicosanoids from tissue, an optimized solvent combination is necessary to cover the whole polarity range of the metabolites, including the polar prostaglandins and the less polar PUFAs. Well-established lipid extraction protocols based on liquid–liquid extraction according to Bligh and Dyer or Folch [15–18] are limited because of the distribution of PUFAs and eicosanoids in both the water-rich upper layer and the chloroform-rich lower layer. Application of ternary solvent combinations including polar as well as nonpolar solvents seems to be a way to overcome these problems [19]. Special requirements for tissue analysis also include the investigation of conditions for a reproducible homogenization of different tissue structures. Protocols for the analysis of selected PUFAs or eicosanoids in brain or liver tissue for subsequent liquid chromatography (LC)–tandem mass spectrometry (MS/MS) analysis have been published [20–32]. In most cases, homogenized samples [20–27, 30] or lyophilized samples [30, 31] were extracted by multiple steps in different organic solvents to cover a broad polarity range. The last step in sample preparation is often manual desalting by solid-phase extraction (SPE) [20–23, 25–30, 32]. Little is known about the preanalytical requirements of eicosanoid analysis in tissue [33, 34] and defined sample pretreatment protocols; a systematic investigation of all influencing and disturbing factors has not yet been done.

The aims of the present study were the development of a common sample pretreatment protocol for simultaneous PUFA and eicosanoid extraction from small amounts of mouse liver and brain tissue and an investigation of the PUFA and eicosanoid distribution in the distinct liver areas and brain substructures.

## Materials and methods

### Chemicals and reagents

The solvents *n*-hexane, 2-propanol (*i*PrOH), formic acid (FA), acetonitrile (ACN), and methanol (MeOH) of ultrahigh-performance LC/MS grade were purchased from Biosolve (Valkenswaard, Netherlands). Water was produced in-house with a Barnstead GenPure System from Thermo Scientific (Waltham, MA, USA). Methyl *tert-*butyl ether (MTBE) of pro analysis quality was obtained from Carl Roth (Karlsruhe, Germany). 2-Butanol of extra-pure quality was purchased from Thermo Fisher Scientific (Waltham, MA, USA). 2,6-Di-*tert*-butyl-4-methylphenol (BHT) of gas chromatography quality and EDTA of ACS reagent grade were purchased from Sigma-Aldrich (St Louis, MO, USA). Ethyl acetate and chloroform (CHCl_3_) of LC grade, zinc sulfate heptahydrate of pro analysis quality, and phosphate-buffered saline (PBS) of liquid, sterile-filtered, suitable-for-cell-culture grade were obtained from Merck Chemicals (Darmstadt, Germany). Tris (hydroxymethyl)aminomethane–HCl buffer was obtained from Bioanalytic (Umkirch, Germany). Unlabeled and deuterium-labeled PUFA and eicosanoid standards were purchased from Cayman Chemicals (Ann Arbor, MI, USA); the detailed information can be found in Table S1.

### Mouse sample materials

Liver and brain of male wild-type C57BL/6J mice were removed directly after they had been killed, and brain was rinsed in PBS. Cortex, cerebellum, hypothalamus, and hippocampus were dissected carefully and immediately. All samples collected were snap-frozen in liquid nitrogen and stored at −80 °C until further analysis.

### Optimization of tissue extraction protocol

#### Sample rinsing

To remove adhering blood, liver samples (30–70 mg) were rinsed six to nine times in 1 mL PBS by 30 s mixing, followed by centrifugation for 5 min at 600*g* and 4 °C. The PBS washing solutions were centrifuged for 10 min at 4 °C and 13,000*g*. Then 100 μL supernatant was transferred into a measuring cuvette and 1 mL tris(hydroxymethyl)aminomethane–HCl buffer solution was added to determine the free hemoglobin content. Spectrophotometric analysis was performed in triplicate according to the method of Harboe [35] with a SPECORD® 50 PLUS instrument (Analytik Jena, Jena, Germany). Additionally, to evaluate analyte losses during sample rinsing, 200 μL PBS supernatant was added to 50 μL internal standard (IS) mix containing deuterated PUFAs (50 ng/mL) and eicosanoids (5 ng/mL) and 400 μL precipitation solution according to our previously published protocol [36]. After centrifugation, the supernatant was transferred for LC–MS/MS analysis of PUFAs and eicosanoids.

#### Homogenization and storage

For tissue homogenization a Mikro-Dismembrator S® (Sartorius, Göttingen, Germany, 1800 rpm), a Tissue Lyser II® (Qiagen, Venlo, Netherlands; 2500 rpm), and a UCD-300-Bioruptor® next-generation system (Diagenode, Liège, Belgium; 320 W) were compared for both tissue types, liver and brain. Homogenization was performed in 650 μL *n*-hexane/*i*PrOH (60:40 v/v) containing BHT at 50 μg/mL. As recommended by the manufacturers, steel balls (three per 100 mg tissue) were added using the ball mills. Homogenization was performed in 1.5-mL safe-lock polypropylene tubes for 5 min at 4 °C and at the highest device performance setting for all devices (Mikro-Dismembrator S® 1800 rpm, Tissue Lyser II® 2500 rpm, UCD-300-Bioruptor® 320 W). The resulting homogenate was examined microscopically with 20-fold magnification with use of a Primovert microscope (Carl Zeiss Microscopy, Jena, Germany). Homogenates representing 1 mg liver tissue were transferred into a new 1.5-mL safe-lock polypropylene tube. For comparison, homogenates remaining in the homogenization solvent and homogenates dried by gentle evaporation under a nitrogen stream (10 °C, 5 min) until dryness were stored for 3 and 30 days at −80 °C. Samples were extracted in *n*-hexane/*i*PrOH (60:40 v/v) for 1 h at 4 °C and 600 rpm with a ThermoMixer (Eppendorf, Hamburg, Germany). PUFAs and eicosanoids were analyzed at 0, 3, and 30 days (three replicates from one pool sample) to investigate the sample stability.

#### Extraction conditions

Ten solvent combinations, listed in Table S2, were tested to optimize the extraction process according to the number and reproducibility of extracted analytes, manual effort, and feasibility as well as in vitro changes of the eicosanoid composition. Dried liver aliquots (*n* = 3) representing 1 mg tissue were mixed with 600 μL extraction solvent and 50 μL IS mix. The extraction was performed in a 1.5 mL safe-lock polypropylene tube with use of a at 600 rpm. The extraction temperature was evaluated at 4, 20, and 37 °C for one extraction cycle and an extraction time of 1 h. The number of extraction cycles (one, two, and three cycles) was investigated at 4 °C and for an extraction time of 1 h. The cycle time was investigated up to 24 h (one cycle) at 4 °C. Each extraction cycle corresponds to 1 h extraction with fresh solvent; extracts from multiple cycles were combined. Addition of BHT (50 μg/mL), EDTA (100 μM), and FA (0.01%, 0.1%, and 1%) was studied. The effect of tissue amount was compared for aliquots representing 1, 3, and 5 mg tissue. All experiments were performed with three replicates of the same liver pool sample.

#### Recovery and variability

The recovery experiment was performed in the extracted 1 mg liver and brain samples by comparing the peak areas of the IS after sample extraction and a blank solution containing the same amount of IS without extraction. The experiments were performed with three replicates of one liver pool sampleand one brain pool sample. For potential matrix interference, the IS recovery was determined in 5-mg tissue homogenates used for extraction. Variability was assessed with a homogenized pooled liver sample. Liver aliquots (1 mg) were extracted according to the optimized protocol. Within-run variability was determined by measuring a pooled liver sample ten times in one run. Between-run variability was calculated by measuring the pooled liver sample on ten consecutive working days. Coefficients of variation were calculated as the ratio of the standard deviation to the mean.

### Distribution of PUFAs and eicosanoids in mouse liver and brain

The experiments were performed with three replicates of one liver pool sample and one brain pool sample. A whole mouse liver was dissected into left lateral, left medial, right lateral, right medial, and caudate lobes. Tissue samples weighing about 30–70 mg were prepared (*n* = 8). According to the optimized protocol, the liver samples were rinsed four times in 1 mL PBS to remove residual blood before homogenization.

Mouse brain was rinsed in PBS. Subsequently, cerebellum, hypothalamus, cortex, and hippocampus were dissected. The individual brain substructures of six mice, differing in size and weight, were pooled (cerebellum 122 mg, hypothalamus 56 mg, cortex 49 mg, and hippocampus 11 mg). Homogenization was performed in 650 μL *n*-hexane/*i*PrOH (60/40 v/v) containing BHT at 50 μg/mL. The solvent was dried for 5 min by a gentle nitrogen stream at 10 °C. Dried aliquots representing 1 mg tissue were extracted by adding 600 μL *n*-hexane/*i*PrOH (60/40 v/v) containing 0.1% FA and 50 μL IS solution for . After centrifugation (10 min, 4 °C, 1000*g*), the supernatant was transferred into a new 1.5-mL polypropylene tube, the solvent was evaporated again under a nitrogen stream until dryness, and was stored at −80 °C until further analysis. Before analysis, the dried sample was reconstituted in 500 μL of the starting LC eluent MeOH/water (62:38 v/v), followed by 30 min mixing and 10 min centrifugation (10,000*g*) at 4 °C. The supernatant was transferred into an autosampler vial for LC–MS/MS analysis.

### LC–MS/MS analysis

The targeted LC–MS/MS analyses for quantification of 7 PUFAs and 94 eicosanoids were performed according to our previously published method [36]. The LC system consisted of a Prominence ultrafast LC system from Shimadzu (Duisburg, Germany), with two high-pressure gradient pumps (LC-20ADXR), an isocratic pump (LC-20AD), a column oven (CTO-20AC), and a control module (CBM-20A), and an HTS PAL autosampler from CTC Analytics (Zwingen, Switzerland), which was coupled to a QTRAP® 5500 mass spectrometer (SCIEX, Framingham, MA, USA) equipped with a Turbo V™ ion spray source operating in negative ion mode. In the LC system, an online SPE was implemented. The online SPE was performed with a Strata-X extraction column (20 mm × 2.1 mm inner diameter, 25-μm particle size, Phenomenex, Aschaffenburg, Germany) and MeOH/water/FA (10:90:0.02 v/v/v) with a flow rate of 3 mL/min for 1 min. Chromatographic separation was performed on a core-shell LC column (Kinetex C18, 100 × 2.1 mm inner diameter, 2.6-μm particle size, Phenomenex, Aschaffenburg, Germany) with a flow rate of 0.6 mL/min and the gradient as follows: 0% to 90% eluent B in 8 min [eluent A was water/ACN/FA (63:37:0.02 v/v/v), eluent B was *i*PrOH/ACN (50:50 v/v)], 90% eluent B for 2 min, and reequilibration of the columns for 2 min. The column oven was kept at 35 °C.

### Statistical analysis

Data were expressed as the mean ± standard deviation. For statistical data processing, IBM® SPSS® Statistics 20 (IBM Deutschland, Ehningen, Germany) was used. Student’s *t* test was performed for significance testing. Significance levels were determined on the basis of *p* < 0.05, *p* < 0.01, or *p* < 0.001.

## Results

### Sample rinsing

Quantitative data regarding the free hemoglobin, PUFA, and eicosanoid concentrations in the six PBS rinsing fractions of the liver tissue are presented in Table S3. Only five eicosanoids (prostaglandin F_2α_, 8,9-dihydroxyeicosatrienoic acid (8,9-DHET), 13-hydroxyoctadecadienoic acid (13-HODE), 15-hydroxyeicosatetraenoic acid (15-HETE), 15-oxoeicosatetraenoic acid (15-oxo-ETE), and arachidonic acid (ARA) were detectable in all six rinsing fractions. In fraction 4, the lowest peak areas were found for all the lipids measured. 8,9-DHET, 13-HODE, 15-HETE, and ARA remained stable in fractions 4–6 at about 0.9–2.1% of the peak areas in the liver extracts. Since brain tissue had already been rinsed before dissection into substructures, no additional rinsing step was necessary before homogenization.

### Homogenization and storage

For liver tissues, different homogenate particle sizes were observed depending on the homogenization device. As shown in Fig. S1, Tissue Lyser II® produced a homogenate with a particle size of about 40 μm. Mikro-Dismembrator S® generated a particle size of 20 μm. The finest homogenate, with 5-μm particle size, was obtained with the ultrasonic device UCD-300-Bioruptor®. For brain tissues, all three homogenizers produced a fine dispersion with a particle size smaller than 5 μm. The addition of BHT at 50 μg/mL during homogenization for 5 min at 4 °C did not significantly affect the results of PUFA and eicosanoid analysis (data not shown).

In Table S4, the differences between liquid storage and storage of dried homogenate at −80 °C are expressed as the relative deviation to time point 0 (direct analysis after homogenization). After storage for 3 days, up to 36% higher eicosanoid concentrations were observed in the solvent-containing samples compared with dried samples. Dried samples were found to be stable for up to 30 days of storage, with the exception of prostaglandin D_3_, prostaglandin E_3_, and 15-oxo-ETE.

### Extraction conditions

The extractions using ten different solvent combinations were investigated by comparison of peak areas after extraction of 1 mg dried liver homogenate for 1 h at 4 °C. In Table 1, the peak areas of the detected analytes for *n*-hexane/*i*PrOH (60:40 v/v) extraction are set to 100%, and relative differences in peak areas of the solvent combinations are provided. The *n*-hexane/*i*PrOH/MeOH (60:20:20 v/v/v), *n*-hexane/ethyl acetate (60:40 v/v), and *n*-hexane/ethyl acetate/MeOH (60:20:20 v/v/v) solvent systems proved to be unsuitable because of the formation of a two-phase system during extraction. MTBE/*i*PrOH (60:40 v/v), MTBE/*i*PrOH/MeOH (60:20:20 v/v/v), and CHCl_3_/MeOH (50:50 v/v) were unsuitable because of low extraction of HETEs, epoxyeicosatrienoic acids (EETs), and PUFAs. Ethyl acetate/*i*PrOH (60:40 v/v), ethyl acetate/*i*PrOH/MeOH (60:20:20 v/v/v), and the two-step procedure involving 2-butanol/MeOH (75:25 v/v) and *n*-hexane/ethyl acetate (75:25 v/v) showed a high variability for oxo-ETEs and EETs. The best extraction solvent for reproducible extraction with low variability for PUFAs, and especially for the DHETs, HETEs, and HODEs, was *n*-hexane/*i*PrOH (60:40 v/v). No influence of the extraction solvent on the background noise of the mass transitions during LC–MS/MS was observed (Fig. S2).

**Table 1:** Relative comparison of polyunsaturated fatty acids and eicosanoids in mouse liver extracted with different solvent combinations. *n*-hexane/(*i*PrOH) (60:40 v/v) was set at 100%.

	*n*-Hexane/*i*PrOH (60:40 v/v)	*n*-Hexane/*i*PrOH/MeOH (60:20:20 v/v/v)	*n*-Hexane/ethyl acetate (60:40 v/v)	*n*-Hexane/ethyl acetate/MeOH (60:20:20 v/v/v)	MTBE/*i*PrOH (60:40 v/v)	MTBE/*i*PrOH/MeOH (60:20:20 v/v/v)	Ethyl acetate/*i*PrOH (60:40 v/v)	Ethyl acetate/*i*PrOH/MeOH 60:20:20 v/v/v	(1) 2-Butanol/MeOH (75:25 v/v) (2) *n*-hexane/ethyl acetate (5:25 v/v)	CHCl_3_/MeOH (50:50 v/v)
EPA	100 **±** 7	108 **±** 22 n.s.	139 **±** 22*	114 **±** 7 n.s.	106 **±** 24 n.s.	110 **±** 12 n.s.	111 **±** 7 n.s.	116 **±** 10 n.s.	142 **±** 6*	54 **±** 25**
α-/γ-LA	100 **±** 5	115 **±** 23 n.s.	149 **±** 22*	111 **±** 8 n.s.	107 **±** 23 n.s.	114 **±** 14 n.s.	87 **±** 7 n.s.	85 **±** 8 n.s.	101 **±** 6 n.s.	25 **±** 29**
DHA	100 **±** 7	111 **±** 16 n.s.	134 **±** 22*	123 **±** 10 n.s.	112 **±** 21 n.s.	113 **±** 11 n.s.	105 **±** 7 n.s.	114 **±** 5 n.s.	122 **±** 6 n.s.	59 **±** 19***
ARA	100 **±** 5	108 **±** 20 n.s.	138 **±** 26*	115 **±** 6 n.s.	103 **±** 21 n.s.	111 **±** 11 n.s.	110 **±** 7 n.s.	117 **±** 8 n.s.	122 **±** 10 n.s.	92 **±** 11***
LA	100 **±** 4	156 **±** 50 n.s.	134 **±** 26*	111 **±** 5 n.s.	102 **±** 18 n.s.	109 **±** 8 n.s.	103 **±** 9 n.s.	107 **±** 6*	110 **±** 7 n.s.	73 **±** 17***
DHGLA	100 **±** 5	107 **±** 16 n.s.	136 **±** 26*	120 **±** 9*	105 **±** 24 n.s.	120 **±** 20 n.s.	107 **±** 9 n.s.	119 **±** 6 n.s.	139 **±** 1 n.s.	66 **±** 24***
6-Keto-PGF_1α_	100 **±** 1	97 **±** 16 n.s.	35 **±** 4**	105 **±** 6 n.s.	101 **±** 15 n.s.	100 **±** 11 n.s.	95 **±** 3 n.s.	98 **±** 2 n.s.	102 **±** 1 n.s.	102 **±** 15 n.s.
TxB_2_	n.d.	n.d.	n.d.	n.d.	n.d.	n.d.	96 **±** 3	91 **±** 5	100 **±** 14	224 **±** 10
PGF_2α_	100 **±** 3	92 **±** 16 n.s.	96 **±** 10**	101 **±** 11 n.s.	88 **±** 16 n.s.	86 **±** 11 n.s.	105 **±** 9 n.s.	102 **±** 9 n.s.	100 **±** 6 n.s.	114 **±** 19 n.s.
15-Keto PGF_2α_/8-iso-PGE_2_/PGE_2_	n.d.	n.d.	n.d.	n.d.	n.d.	n.d.	70 **±** 15	92 **±** 13	100 **±** 9	127 **±** 13
15-Keto PGE_2_/8-iso-15-Keto PGE_2_	n.d.	n.d.	n.d.	n.d.	n.d.	n.d.	n.d.	n.d.	100 **±** 43	n.d.
LTB_4_	100 **±** 20	93 **±** 13 n.s.	112 **±** 16 n.s.	95 **±** 8 n.s.	69 **±** 11*	71 **±** 10*	n.d.	n.d.	n.d.	n.d.
5,6-DHET	100 **±** 10	103 **±** 16 n.s.	121 **±** 14 n.s.	105 **±** 12 n.s.	120 **±** 17 n.s.	114 **±** 11 n.s.	125 **±** 3 n.s.	122 **±** 5 n.s.	139 **±** 2 n.s.	99 **±** 18 n.s.
8,9-DHET	100 **±** 5	103 **±** 17 n.s.	114 **±** 13 n.s.	104 **±** 3 n.s.	90 **±** 20 n.s.	92 **±** 15 n.s.	104 **±** 7 n.s.	109 **±** 7 n.s.	113 **±** 8 n.s.	107 **±** 9 n.s.
11,12-DHET	100 **±** 6	105 **±** 17 n.s.	119 **±** 11 n.s.	114 **±** 3*	101 **±** 23 n.s.	98 **±** 10 n.s.	112 **±** 4 n.s.	117 **±** 3 n.s.	123 **±** 3 n.s.	99 **±** 11 n.s.
14,15-DHET	100 **±** 6	101 **±** 15 n.s.	116 **±** 10 n.s.	109 **±** 4 n.s.	99 **±** 21 n.s.	95 **±** 10 n.s.	105 **±** 5 n.s.	116 **±** 5 n.s.	145 **±** 1 n.s.	104 **±** 11 n.s.
12-HHT	100 **±** 12	115 **±** 18 n.s.	117 **±** 15 n.s.	124 **±** 4 n.s.	115 **±** 26 n.s.	107 **±** 8 n.s.	78 **±** 10 n.s.	101 **±** 4 n.s.	120 **±** 1 n.s.	65 **±** 14*
12-HEPE	100 **±** 14	115 **±** 21 n.s.	128 **±** 14 n.s.	116 **±** 2 n.s.	108 **±** 22 n.s.	102 **±** 5 n.s.	84 **±** 2 n.s.	98 **±** 10 n.s.	125 **±** 4 n.s.	86 **±** 27 n.s.
5-HETE	100 **±** 16	92 **±** 19 n.s.	102 **±** 11*	114 **±** 10**	78 **±** 7 n.s.	76 **±** 7 n.s.	85 **±** 7 n.s.	97 **±** 17 n.s.	89 **±** 6 n.s.	82 **±** 23 n.s.
8-HETE	100 **±** 12	97 **±** 17 n.s.	109 **±** 11 n.s.	106 **±** 10 n.s.	94 **±** 21 n.s.	93 **±** 3 n.s.	93 **±** 11 n.s.	110 **±** 5 n.s.	136 **±** 1 n.s.	n.d.
9-HETE	100 **±** 9	84 **±** 10 n.s.	107 **±** 13*	106 **±** 11 n.s.	88 **±** 13 n.s.	75 **±** 13 n.s.	186 **±** 7*	224 **±** 10*	289 **±** 1***	92 **±** 15*
11-HETE	100 **±** 5	113 **±** 20 n.s.	126 **±** 12*	123 **±** 4*	101 **±** 17 n.s.	99 **±** 2 n.s.	116 **±** 9 n.s.	134 **±** 5 n.s.	163 **±** 2*	95 **±** 18 n.s.
12-HETE	100 **±** 11	98 **±** 16 n.s.	108 **±** 11 n.s.	106 **±** 9 n.s.	94 **±** 21 n.s.	94 **±** 2 n.s.	94 **±** 11 n.s.	109 **±** 7 n.s.	133 **±** 3 n.s.	99 **±** 25 n.s.
15-HETE	100 **±** 10	104 **±** 21 n.s.	115 **±** 9 n.s.	136 **±** 12 n.s.	95 **±** 12 n.s.	97 **±** 1**	110 **±** 8 n.s.	125 **±** 6 n.s.	142 **±** 6**	105 **±** 27 n.s.
16-HETE	100 **±** 6	114 **±** 20 n.s.	116 **±** 12 n.s.	140 **±** 11*	110 **±** 16 n.s.	111 **±** 5*	75 **±** 2 n.s.	89 **±** 4*	100 **±** 6 n.s.	95 **±** 21 n.s.
17-HETE	100 **±** 8	116 **±** 20 n.s.	94 **±** 17 n.s.	118 **±** 14 n.s.	115 **±** 22 n.s.	104 **±** 11 n.s.	n.d.	106 **±** 11 n.s.	104 **±** 6 n.s.	85 **±** 17*
18-HETE	100 **±** 5	107 **±** 21 n.s.	120 **±** 8 n.s.	123 **±** 9*	125 **±** 22 n.s.	113 **±** 11*	98 **±** 0 n.s.	111 **±** 7 n.s.	141 **±** 4 *	114 **±** 17 n.s.
9-HODE	100 **±** 5	92 **±** 17 n.s.	110 **±** 12*	102 **±** 6 n.s.	91 **±** 18 n.s.	89 **±** 6 n.s.	93 **±** 14 n.s.	91 **±** 9 n.s.	96 **±** 3 n.s.	89 **±** 18*
13-HODE	100 **±** 9	124 **±** 42 n.s.	107 **±** 11 n.s.	105 **±** 7 n.s.	91 **±** 13 n.s.	88 **±** 2 n.s.	92 **±** 10 n.s.	91 **±** 4 n.s.	102 **±** 6 n.s.	104 **±** 16 n.s.
5-oxo-ETE	100 **±** 56	149 **±** 41 n.s.	73 **±** 9 n.s.	297 **±** 13 *	n.d.	83 **±** 14 n.s.	n.d.	106 **±** 37 n.s.	206 **±** 27*	146 **±** 31 n.s.
12-oxo-ETE	100 **±** 26	104 **±** 66 n.s.	127 **±** 28 n.s.	90 **±** 17 n.s.	40 **±** 7**	42 **±** 5*	72 **±** 17*	179 **±** 52 n.s.	129 **±** 36 n.s.	63 **±** 43 n.s.
15-oxo-ETE	n.d.	n.d.	n.d.	n.d.	n.d.	n.d.	45 **±** 13	76 **±** 39	100 **±** 25	245 **±** 65
5,6-EET	n.d.	n.d.	n.d.	n.d.	n.d.	n.d.	n.d.	94 **±** 21	100 **±** 33	n.d.
8,9-EET	100 **±** 46	110 **±** 24 n.s.	238 **±** 14 n.s.	298 **±** 26*	83 **±** 15 n.s.	71 **±** 6 n.s.	148 **±** 17 n.s.	176 **±** 12 n.s.	373 **±** 14**	88 **±** 51 n.s.
11,12-EET	100 **±** 52	100 **±** 17 n.s.	955 **±** 2 n.s.	384 **±** 36*	84 **±** 2 n.s.	71 **±** 7 n.s.	261 **±** 18 n.s.	233 **±** 34 n.s.	353 **±** 2*	59 **±** 36**
14,15-EET	100 **±** 43	58 **±** 3 n.s.	1684 **±** 3 n.s.	184 **±** 31 n.s.	63 **±** 14 n.s.	66 **±** 11 n.s.	319 **±** 42 n.s.	262 **±** 65 n.s.	230 **±** 19*	50 **±** 27**
5-HpETE	100 **±** 51	147 **±** 77 n.s.	136 **±** 25 n.s.	106 **±** 36 n.s.	n.d.	n.d.	86 **±** 25 n.s.	160 **±** 24 n.s.	267 **±** 30 n.s.	153 **±** 124 n.s.

Student's *t* test: *p ≤ 0.05, **p ≤ 0.01, ***p ≤ 0.001; n.s. not significant; relative to *n*-hexane/*i*PrOH (60:40 v/v)

*ARA* arachidonic acid, *DHA* docosahexaenoic acid, *DHET* dihydroxyeicosatrienoic acid, *DHGLA* dihomo-γ-linolenic acid, *EET* epoxyeicosatrienoic acid, *EPA* eicosapentaenoic acid, *ETE* eicosatetraenoic acid, *HEPE* hydroxyeicosapentaenoic acid, *HETE* hydroxyeicosatetraenoic acid, *HHT* hydroxyheptadecatrienoic acid, *HODE* hydroxyoctadecadienoic acid, *HpETE* hydroperoxyeicosatetraenoic acid, *LA* linoleic acid, *α-/γ-LA α-/*γ-linolenic acid *LTB*_*4*_ leukotriene B_4_, *MeOH* methanol, *MTBE* methyl *tert*-butyl ether, n.d. not determined, *PG* prostaglandin, *TxB*2 thromboxane B_2_

The investigation of extraction conditions for *n*-hexane/*i*PrOH (60:40 v/v) revealed a tendency for lower PUFA and eicosanoid concentrations with increased extraction temperatures of 20 and 37 °C compared with 4 °C (Table S5a). A single extraction of 1 mg homogenate using 650 μL *n*-hexane/*i*PrOH (60:40 v/v) at 4 °C for 1 h was found to be sufficient for the extraction of PUFAs and eicosanoids and showed high reproducibility (Table S5b). PUFA concentrations were not affected by longer extraction time or multiple extraction cycles and remained at (100 ± 10)%. After three repetitive extraction cycles with fresh solvent for 1 h at 4 °C, elevated concentrations of eicosanoids formed by autoxidation, for example, 12-oxo-ETE (+105%) and 14,15-EET (+177%), were observed. A prolonged extraction time of 24 h did not improve the extraction compared with 1 h, with few exceptions, for example, 12-oxo-ETE (+47%), and 14,15-EET (−34%).

**Fig. 1:**
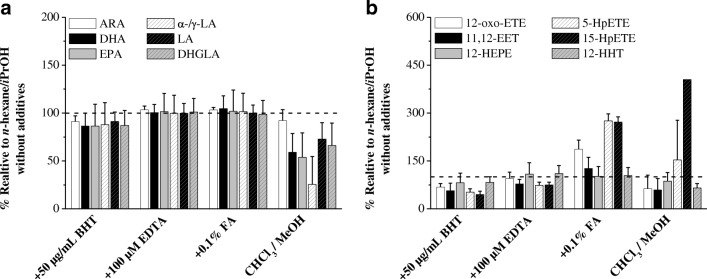
Comparison of polyunsaturated fatty acids (**a**) and eicosatetraenoic acids (ETEs), 11,12-epoxyeicosatrienoic acid (11,12-EET), 12-hydroxyeicosapentaenoic acid (12-HEPE), and 12-hydroxyheptadecatrienoic acid (12-HHT) (**b**) in mouse liver extracted with *n*-hexane/2-propanol (*i*PrOH) (60:40 v/v) with the addition of 2,6-di-tert-butyl-4-methylphenol (BHT), EDTA, and formic acid CHCl_3_ (FA) and extracted with cholorform/methanol (CHCl_3_ MeOH) without additives. Changes are presented as relative to the extraction with *n*-hexane/*i*PrOH (60:40 v/v) without additives, which was set as 100% (dashed line). ARA arachidonic acid, DHGLA dihomo-γ-linolenic acid, DHA docosahexaenoic acid, EPA eicosapentaenoic acid, HpETE hydroperoxyeicosatetraenoic acid, LA linoleic acid,*α-/γ-LA α-/*γ-linolenic acid.

The additives, such as BHT, EDTA, and FA, were studied for prevention of autoxidation and improvement of extraction (Table S5c). The addition of BHT at 50 μg/mL, serving as an antioxidant, did not show any significant changes during a single extraction cycle of 1 h with *n*-hexane/*i*PrOH (60:40 v/v) at 4 °C. A slight but not significant increase in signal intensity was observed on addition of 100 μM EDTA. The addition of FA during extraction results in an increased concentration of most metabolites. Significantly higher concentrations for prostaglandins (up to 152%), especially hydroperoxyeicosatetraenoic acids (HpETEs) (up to +469%), and EETs (up to +464%), were observed on addition of 1% FA to *n*-hexane/*i*PrOH (60:40 v/v). The influence of additives is exemplarily shown for extraction of PUFAs, HpETEs, and EETs in Fig. 1. The IS was not affected by the additives and did not show similar signal enhancement on FA addition (data not shown).

**Fig. 2:**
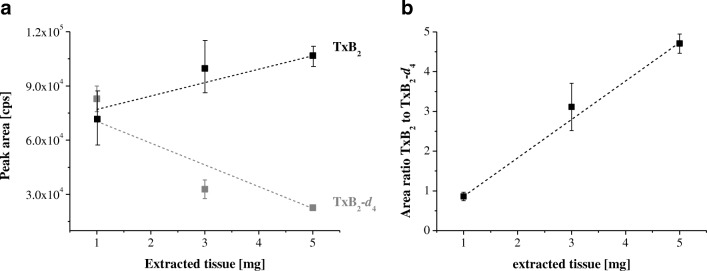
**a** Peak areas of thromboxane B_**2**_ (TxB_2_) and TxB_2_-*d*_4_ versus amount of extracted liver tissue. **b** Area ratio of TxB_2_ to TxB_2_-*d*_4_ versus amount of extracted tissue

**Fig. 3:**
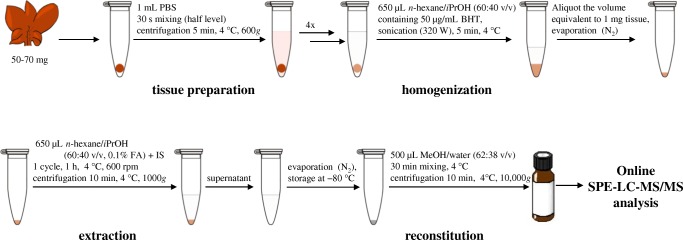
Final sample preparation and extraction protocol for the liquid chromatography–tandem mass spectrometry (LC–MS/MS) analysis of polyunsaturated fatty acids and eicosanoids in soft tissue. BHT 2,6-di-*tert*-butyl-4-methylphenol, FA formic acid, *i*PrOH 2-propanol, IS internal standard, SPE solid-phase extraction

Finally, the amount of homogenate was increased from 1 to 5 mg to improve the detection of less abundant eicosanoids. Signal suppression was observed with increasing amounts of extracted liver tissue, exemplarily shown for thromboxane B_2_ (TxB_2_) in Fig. 2. The signal of the IS (TxB_2_-*d*_4_) decreased with increasing tissue amount. Simultaneously, the analyte (TxB_2_) signal increased, but was not proportional to the increase in tissue amount (Fig. 2a). However, this effect is not apparent regarding the area ratio of TxB_2_ to TxB_2_-*d*_4_ (Fig. 2b). A proportional slope was observed for the area ratio. Nevertheless, signal suppression was observed for all ISs in the range between 29% and 74% in extracts of 5 mg compared with 1 mg of the same liver tissue. Because of the limited availability of distinct tissue and the observed effect of ion suppression, we recommend the extraction of 1 mg tissue. The final conditions for sample pretreatment are summarized in Fig. 3.

### Recovery and variability

The relative IS recovery in extracted samples of 1 mg liver and brain compared with a blank solution is given in Table 2. The IS recovery was 45–146% for liver and 45–149% for brain. Within-run and between-run variability ranged between 7% and 18% for PUFAs and between 1% and 24% for eicosanoids. The between-run variability was 7–13% for PUFAs and 1–24% for eicosanoids. Increasing the tissue amount to 5 mg resulted in higher matrix interferences with decreased IS signals (data not shown).

**Table 2:** Polyunsaturated fatty acid (PUFA) and eicosanoid validation data: Within-run variability and between-run variability (n = 10) were determined in 1-mg aliquots of the same pooled liver sample. The relative internal standard (IS) recovery, compared with a blank solution, was determined in samples of 1 mg liver and 1 mg brain. Samples were extracted in *n*-hexane/2-propanol (60:40 v/v) containing 0.1% formic acid (1 h, 4 °C, 600 rpm) and subsequently analyzed by liquid chromatography–tandem mass spectrometry.

Analyte		Within-run variability (*n* = 10)		Between-run variability (*n* = 10)		Corresponding IS	IS recovery (%)	
		Mean ± SD	CV (%)	Mean ± SD	CV (%)		1 mg liver	1 mg brain
PUFAs (pg/mg)	LA	4.72 × 10^5^ ± 6.09 × 10^4^	13	4.91 × 10^5^ ± 3.51 × 10^4^	7	ARA-*d*_8_	111	119
	EPA	2.83 × 10^4^ ± 3.68 × 10^3^	13	3.23 × 10^4^ ± 2.40 × 10^3^	7	EPA-*d*_5_	114	101
	DHA	1.99 × 10^5^ ± 2.83 × 10^4^	14	2.05 × 10^5^ ± 1.55 × 10^4^	8	DHA-*d*_5_	117	149
	ARA	2.70 × 10^5^ ± 3.41 × 10^4^	13	2.68 × 10^5^ ± 1.97 × 10^4^	7	ARA-*d*_8_	111	119
	DHGLA	5.57 × 10^4^ ± 1.00 × 10^4^	18	4.90 × 10^4^ ± 6.32 × 10^3^	13	ARA-*d*_8_	111	119
	α-/γ-LA	3.84 × 10^4^ ± 2.66 × 10^3^	7	4.09 × 10^4^ ± 2.76 × 10^3^	7	EPA-*d*_5_	114	101
Eicosanoids (fg/mg)	LA-derived metabolites							
	9-HODE	1.81 × 10^6^ ± 1.86 × 10^5^	10	1.72 × 10^6^ ±1.30 × 10^5^	8	13(*S*)-HODE-*d*_4_	83	85
	13-HODE	2.17 × 10^6^ ± 2.62 × 10^5^	12	2.07 × 10^6^ ± 1.87 × 10^5^	9	13(*S*)-HODE-*d*_4_	83	85
	EPA-derived metabolites							
	12-HEPE	1.60 × 10^6^ ± 1.93 × 10^5^	12	1.76 × 10^6^ ± 2.09 × 10^5^	12	15(*S*)-HETE-*d*_8_	84	83
	ARA-derived metabolites							
	TxB_2_	2.23 × 10^5^ ± 3.82 × 10^4^	17	2.16 × 10^5^ ± 3.99 × 10^4^	18	TxB_2_-*d*_4_	101	87
	6-Keto-PGF_1α_	1.22 × 10^5^ ± 1.33 × 10^4^	11	1.27 × 10^5^ ± 1.74 × 10^4^	14	PGF_2α_-*d*_4_	146	123
	PGF_2α_	2.62 × 10^5^ ± 3.72 × 10^4^	14	2.76 × 10^5^ ± 3.00 × 10^4^	11	PGF_2α_-*d*_4_	146	123
	15-Keto PGF_2α_/8-iso-PGE_2_/PGE_2_	2.83 × 10^5^ ± 1.89 × 10^3^	1	2.81 × 10^5^ ± 1.15 × 10^3^	1	PGE_2_-*d*_4_	120	92
	8-Iso-15-keto-PGF_2α_	2.07 × 10^4^ ± 4.87 × 10^3^	24	2.03 × 10^4^ ± 4.92 × 10^3^	24	8-Iso-PGF_2α_-*d*_4_	143	116
	PGD_2_	1.60 × 10^4^ ± 3.87 × 10^3^	24	1.70 × 10^4^ ± 2.92 × 10^3^	17	PGD_2_-*d*_4_	88	63
	15-Deoxy-Δ^12,14^-PGD_2_	1.98 × 10^4^ ± 6.02 × 10^2^	3	1.92 × 10^4^ ± 6.99 × 10^2^	4	PGD_2_-*d*_4_	88	63
	12-HHT	1.30 × 10^6^ ± 1.29 × 10^5^	10	1.36 × 10^6^ ± 7.31 × 10^4^	5	15(*S*)-HETE-*d*_8_	84	83
	5-HETE	1.08 × 10^5^ ± 7.26 × 10^3^	7	1.15 × 10^5^ ± 1.46 × 10^4^	13	5(*S*)-HETE-*d*_8_	74	78
	8-HETE	3.91 × 10^6^ ± 3.91 × 10^5^	10	3.98 × 10^6^ ± 3.12 × 10^5^	8	15(*S*)-HETE-*d*_8_	84	83
	11-HETE	2.55 × 10^5^ ± 2.91 × 10^4^	11	2.69 × 10^5^ ± 2.20 × 10^4^	8	15(*S*)-HETE-*d*_8_	84	83
	12-HETE	4.33 × 10^6^ ± 4.27 × 10^5^	10	4.44 × 10^6^ ± 3.78 × 10^5^	9	5(*S*)-HETE-*d*_8_	74	78
	15-HETE	2.41 × 10^5^ ± 2.56 × 10^4^	11	2.45 × 10^5^ ± 2.69 × 10^4^	11	15(*S*)-HETE-*d*_8_	84	83
	18-HETE	4.29 × 10^4^ ± 5.03 × 10^3^	12	4.29 × 10^4^ ± 2.76 × 10^3^	6	15(*S*)-HETE-*d*_8_	84	83
	8,9-DHET	6.66 × 10^4^ ± 5.83 × 10^3^	9	6.86 × 10^4^ ± 3.88 × 10^3^	6	(±)-8,9-DHET-*d*_11_	57	50
	11,12-DHET	1.51 × 10^5^ ± 1.31 × 10^4^	9	1.48 × 10^5^ ± 6.02 × 10^3^	4	(±)-8,9-DHET-*d*_11_	57	50
	11,12-EET	3.81 × 10^4^ ± 2.68 × 10^3^	7	3.58 × 10^4^ ± 5.41 × 10^3^	15	(±)-8,9-DHET-*d*_11_	57	50
	5-HpETE	n.d.		n.d.		5(S)-HETE-*d*_8_	74	78
	12-HpETE	n.d.		n.d.		5(S)-HETE-*d*_8_	74	78
	15-HpETE	n.d.		n.d.		5(S)-HETE-*d*_8_	74	78
	12-oxo-ETE	2.82 × 10^4^ ± 1.06 × 10^3^	4	2.85 × 10^4^ ± 1.57 × 10^3^	6	5-Oxo-ETE-*d*_7_	45	45

*ARA* arachidonic acid, *CV* coefficient of variation, *DHA* docosahexaenoic acid, *DHET* dihydroxyeicosatrienoic acid, *DHGLA* dihomo-γ-linolenic acid, *EET* epoxyeicosatrienoic acid, *EPA* eicosapentaenoic acid, *ETE* eicosatetraenoic acid, *HEPE* hydroxyeicosapentaenoic acid, *HETE* hydroxyeicosatetraenoic acid, *HHT* hydroxyheptadecatrienoic acid, *HODE* hydroxyoctadecadienoic acid, *HpETE* hydroperoxyeicosatetraenoic acid, *LA* linoleic acid,* α-/γ-LA α-/*γ-linolenic acid *n.d.* not determined, *PG* prostaglandin, *SD* standard deviation, *TxB*2 thromboxane B_2_

### Distribution of PUFAs and eicosanoids in mouse liver and brain

**Fig. 4:**
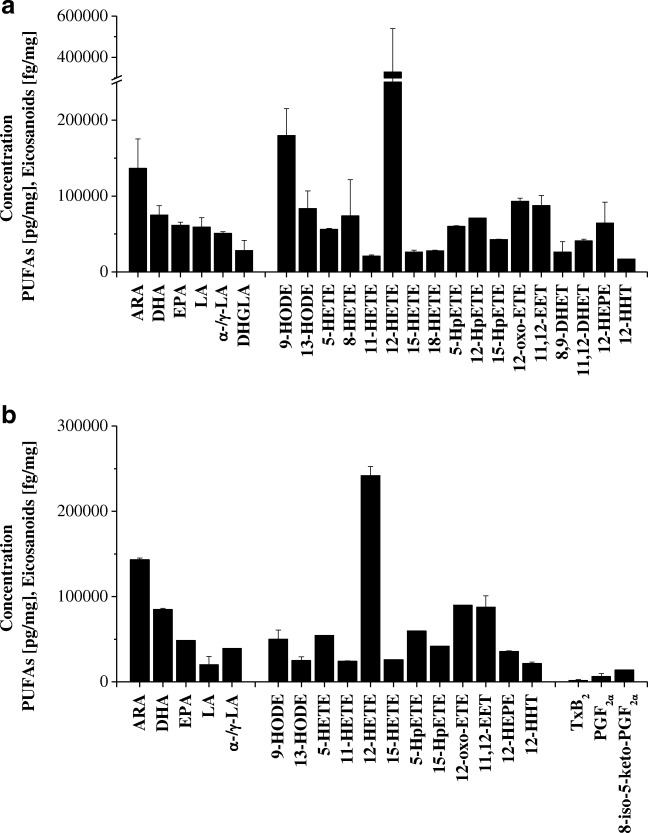
Distribution of polyunsaturated fatty acids (PUFAs) and eicosanoids in the liver (**a**) and the cerebellum (**b**) of C57BL/6J mice. ARA arachidonic acid, DHA docosahexaenoic acid, DHET dihydroxyeicosatrienoic acid, DHGLA dihomo-γ-linolenic acid, EET epoxyeicosatrienoic acid, EPA eicosapentaenoic acid, ETE eicosatetraenoic acid, HEPE hydroxyeicosapentaenoic acid, HETE hydroxyeicosatetraenoic acid, HHT hydroxyheptadecatrienoic acid, HODE hydroxyoctadecadienoic acid, HpETE hydroperoxyeicosatetraenoic acid, LA linoleic acid, *α-/γ-LA α-/*γ-linolenic acid. PGF_2α_ prostaglandin F_2α_, TxB_2_thromboxane B_2_

The region-specific PUFA and eicosanoid distributions of mouse liver (*n* = 6) and brain (*n* = 6) were investigated with the newly developed tissue pretreatment protocol. In liver, 7 PUFAs and 15 eicosanoids were quantified, showing a uniform tissue distribution as given in Table S6. Mainly ARA-derived 12-hydroxyheptadecatrienoic acid (HHT), HETEs, DHETs, and HpETEs were identified. The eicosapentaenoic acid-derived metabolite 12-hydroxyeicosapentaenoic acid and the linoleic acid LA-derived metabolites 9-HODE and 13-HODE were also detected (Table S7). The most prominent eicosanoids were 12-HETE and 9-HODE. The 12-HETE concentration was fourfold to 16-fold higher than the concentrations of the other HETEs detected. The concentration of 9-HODE was twice as high as that of 13-HODE. Thromboxanes and prostaglandins were not detected (Fig. 4a).

**Fig. 5:**
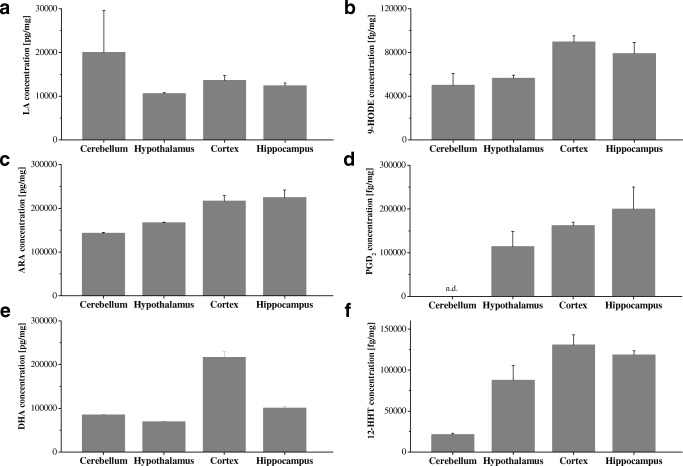
Distribution of polyunsaturated fatty acids **a** linoleic acid (LA), **c** arachidonic acid (ARA), and **e** docosahexaenoic acid (DHA) and eicosanoids **b** 9-hydroxyoctadecadienoic acid (9-HODE), **d** prostaglandin D_**2**_ (PGD_2_), and f 12-hydroxyheptadecatrienoic acid (12-HHT) in brain substructures of C57BL/6J mice. n.d. not detected

In mouse brain compared with liver, we found a different PUFA and eicosanoid distribution as shown in Fig. 4. Mainly the ARA-derived COX metabolites TxB_2_, prostaglandins, 12-HHT, HETEs, 11,12-EET, HpETEs, and 12-oxo-ETE, the eicosapentaenoic acid-derived metabolite 12-hydroxyeicosapentaenoic acid, the linoleic acid-derived metabolites 9-HODE, and 13-HODE, and the dihomo-γ-linolenic acid-derived metabolite prostaglandin F_1α_ (Table S7) were found. Region-specific eicosanoid distributions were identified in brain tissues, as shown in Fig. 5 for selected analytes. Whereas ARA and docosahexaenoic acid (DHA) were the main PUFAs in all four brain regions studied, the major prostaglandins in cortex, hypothalamus, and hippocampus were prostaglandin F_2α_ and prostaglandin D_2_. In contrast, the cerebellum contained only prostaglandin F_2α_. The other eicosanoid concentrations, such as those of HETEs and HODEs, significantly differed within the brain regions. As shown in Table S7, they differed by up to 40-fold. 12-HETE was the prominent HETE in the brain, present at fourfold to tenfold higher concentrations than 5-HETE, 11-HETE, and 15-HETE. Compared with the concentrations of 12-HETE in the cerebellum and the hypothalamus, the concentrations were two to three times higher in the hippocampus and the cortex. The cerebellum contained the lowest amounts of 12-HHT, fourfold to sixfold lower than in the hypothalamus, the hippocampus, and the cortex. The concentrations of HODEs were twofold to fourfold lower in the brain compared with the liver. The concentration of 9-HODE was twice that of 13-HODE in all brain areas, showing the highest concentrations in the cortex. Similar concentrations in all brain regions and the liver were found for 12-oxo-ETE, 5-HpETE, and 15-HpETE.

Quantitative data for PUFAs and eicosanoids in mouse liver and the distribution in the mouse cortex, cerebellum, hypothalamus, and hippocampus of 16-week-old male wild-type controls on a C57BL/6J background (*n* = 6) are provided in Table S7.

## Discussion

PUFAs and eicosanoids are biologically active lipid mediators that play a critical role in different pathological processes, such as (neuro)inflammation and degeneration. Little is known about the region-specific distribution of PUFAs and eicosanoids in liver and brain. However, previous blood analyses identified very low eicosanoid/PUFA abundance and enzymatic and autoxidative in vitro formation as being the main challenges in sample pretreatment [37]. These issues also apply to tissue sample preparation.

### Optimization of tissue extraction protocol

The presence of PUFAs and eicosanoids in blood requires rinsing of the tissue, especially of the well-perfused liver. We observed that washing the liver tissue four times with 1 mL PBS removed adhering blood without significant tissue PUFA and eicosanoid loss. However, the prostaglandin F_2α_ and 15-oxo-ETE concentrations in the rinsing solution increased continuously, indicating potential cell damage and oxidation. Furthermore, homogenization of tissue can activate eicosanoid synthesis in vitro. Tissue sonification revealed a very small particle size and particle size distribution of the homogenate without signs of PUFA activation independent of BHT addition. However, to avoid in vitro lipid peroxidation, the addition of BHT during pretreatment and subsequent storage at −80 °C is recommended. We compared ten different solvent combinations over the expected polarity range for their PUFA and eicosanoid extraction. The highest eicosanoid count was detected with use of ethyl acetate/*i*PrOH/MeOH (60:20:20 v/v/v) and the two-step method including first 2-butanol/MeOH (75:25 v/v) extraction and second *n*-hexane/ethyl acetate (75:25 v/v) extraction. However, technical problems in phase separation, greater manual effort, and the formation of oxidized and peroxidized metabolites such as oxo-ETEs and EETs prevented further application of these solvent combinations. The *n*-hexane/*i*PrOH (60:40 v/v) solvent combination showed good phase separation and resulted in low variability for the PUFA and eicosanoid extraction, especially for prostaglandins, DHETs, HETEs, and HODEs. These results confirm previous findings [38, 39]. We showed that a single extraction cycle, with *n*-hexane/*i*PrOH (60:40 v/v) at 4 °C for 1 h, resulted in sufficient extraction efficiency while having low potential for autoxidation because of the shorter extraction time. The shorter extraction time, as opposed to that for the commonly applied overnight tissue PUFA and eicosanoid extraction [40], is due to the efficient homogenization and the resulting large surface area of the homogenate. Acidification by FA addition improves the extraction of the less polar eicosanoids ETEs and EETs [20, 22, 40–42]. A lower pH leads to reduced protein binding, and the free carboxylic acid form allows improved extraction into the organic solvent [41]. However, greater acidification may lead to eicosanoid alteration [43], and therefore an extremely low pH should be avoided [33]. To benefit from the decreased protein binding and the enhanced extraction of the nonionized forms, we recommend merely a slight acidification by adding 0.1% FA, which corresponds to pH 2.6.

For the optimized extraction protocol presented in Fig. 4, the IS recovery was determined to be 45–146% for liver tissue and 45–149% for brain tissue. Within-run and between-run variability ranging between 1% and 24%, demonstrated good method reproducibility and robustness. Increasing the amount of processed tissue to 5 mg resulted in higher matrix interferences and decreased IS signals. However, to a certain extent, the different signal intensities of the analyte and IS can be compensated by use of the area ratios of the analyte to the IS.

### Distribution of PUFAs and eicosanoids in mouse liver and brain

The PUFA and eicosanoid content in liver and brain (cerebellum, hypothalamus, hippocampus, and cortex) was investigated by LC–MS/MS with the newly developed tissue pretreatment protocol. In the liver, 7 PUFAs and 15 eicosanoids were identified, showing similar distributions in all liver fractions. ARA was found to be the primary PUFA and peroxygenase-derived eicosanoids (HETEs, HODEs) were the most abundant eicosanoids. We confirmed previous findings that described the highest abundance for ARA and docosahexaenoic acid(DHA) in the brain [44] and a differing fatty acid distribution between the brain substructures [45].

Significantly higher amounts of prostaglandins, especially prostaglandin F_2α_, were observed in all four brain substructures compared with the liver. Similar findings were reported for cow brains compared with cow livers [22]. The main enzyme catalyzing these reactions is COX, which occurs in three isoforms. Different regional distributions of COX-1 and COX-2 in specific brain regions have been reported [46–49]. COX-2 expression differ significantly between the brain substructures, showing the highest expression in the cortex and the lowest expression in the cerebellum [26, 48, 49]. These findings may explain the lower concentrations of COX-2-related eicosanoids in the cerebellum.

In mice, we confirmed previous results from cow brain and liver tissue [22], with lower 9-HODE and 13-HODE brain tissue concentrations compared with liver tissue concentrations. Primarily 9-HODE, but not 13-HODE, induces proinflammatory cytokine expression [40], which may have a proinflammatory effect in the brain.

## Conclusion

The tissue pretreatment protocol we established is suitable for the investigation of PUFA and eicosanoid distribution by LC–MS/MS in small amounts of soft tissue. The protocol allows simultaneous and reproducible analysis of a broad range of PUFAs and eicosanoids, while preventing autoxidation during sample preparation. In a first study of mouse liver and brain, we observed high regional heterogeneity in the PUFA and eicosanoid distribution within the brain substructures investigated, but not within the liver sections. This stresses the importance of a region-specific investigation into changes of PUFA and eicosanoid metabolism to increase understanding of the physiological and pathophysiological changes during neuroinflammatory and neurodegenerative processes. The application of the protocol for brain and liver can be easily adapted to other soft tissue. The limitations of the protocol, however, are the numerous steps and the great manual effort. Before application in larger studies, automation of the extraction process should be considered.

## Electronic supplementary material

ESM 1 (PPTX 1.06 mb)

ESM 2 (XLSX 16 kb)

ESM 3 (XLSX 17 kb)

ESM 4 (XLSX 52 kb)

ESM 5 (XLSX 19 kb)

ESM 6 (XLSX 18 kb)

ESM 7 (XLSX 20 kb)

## Abbreviations

ACN: Acetonitrile
ARA: Arachidonic acid
BHT: 2,6-Di-*tert*-butyl-4-methylphenol
COX: Cyclooxygenase
DHET: Dihydroxyeicosatrienoic acid
EET: Epoxyeicosatrienoic acid
ETE: Eicosatetraenoic acid
FA: Formic acid
HETE: Hydroxyeicosatetraenoic acid
HHT: Hydroxyheptadecatrienoic acid
HODE: Hydroxyoctadecadienoic acid
HpETE: Hydroperoxyeicosatetraenoic acid
*i*PrOH: 2-Propanol
IS: Internal standard
LC: Liquid chromatography
MeOH: Methanol
MS/MS: Tandem mass spectrometry
MTBE: Methyl *tert-*butyl ether
PBS: Phosphate-buffered saline
PUFA: Polyunsaturated fatty acid
SPE: Solid-phase extraction

## References

[1] Hotamisligil GS (2006). Inflammation and metabolic disorders. Nature.

[2] Hsieh P-S, Jin J-S, Chiang C-F, Chan P-C, Chen C-H, Shih K-C (2009). COX-2-mediated inflammation in fat is crucial for obesity-linked insulin resistance and fatty liver. Obesity.

[3] Boitard C, Etchamendy N, Sauvant J, Aubert A, Tronel S, Marighetto A, Layé S, Ferreira G (2012). Juvenile, but not adult exposure to high-fat diet impairs relational memory and hippocampal neurogenesis in mice. Hippocampus.

[4] Jeon BT, Jeong EA, Shin HJ, Lee Y, Lee DH, Kim HJ, Kang SS, Cho GJ, Choi WS, Roh GS (2012). Resveratrol attenuates obesity-associated peripheral and central inflammation and improves memory deficit in mice fed a high-fat diet. Diabetes.

[5] Martin RE, Bazan NG (1992). Changing fatty acid content of growth cone lipids prior to synaptogenesis. J Neurochem.

[6] Green P, Glozman S, Kamensky B, Yavin E (1999). Developmental changes in rat brain membrane lipids and fatty acids : the preferential prenatal accumulation of docosahexaenoic acid. J Lipid Res.

[7] Joo M, Sadikot RT (2012). PGD synthase and PGD_2_ in immune resposne. Mediators Inflamm.

[8] Cowley TR, Fahey B, O’Mara SM (2008). COX-2, but not COX-1, activity is necessary for the induction of perforant path long-term potentiation and spatial learning in vivo. Eur J Neurosci.

[9] Alix E, Schmitt C, Strazielle N, Ghersi-Egea J-F (2008). Prostaglandin E2 metabolism in rat brain: role of the blood-brain interfaces. Cerebrospinal Fluid Res.

[10] Cazevieille C, Muller A, Meynier F, Dutrait N, Bonne C (1994). Protection by prostaglandins from glutamate toxicity in cortical neurons. Neurochem Int.

[11] Liang X, Wu L, Hand T, Andreasson K (2005). Prostaglandin D2 mediates neuronal protection via the DP1 receptor. J Neurochem.

[12] Birch EE, Garfield S, Hoffman DR, Uauy R, Birch DG (2000). A randomized controlled trial of early dietary supply of long- chain polyunsaturated fatty acids and mental development in term infants. Dev Med Child Neurol.

[13] Jamieson EC, Farquharson J, Logan RW, Howatson AG, Patrick WJA, Weaver LT, Cockburn F (1999). Infant cerebellar gray and white matter fatty acids in relation to age and diet. Lipids.

[14] Biringer RG (2019). The role of eicosanoids in alzheimer’s disease. Int J Environ Res Public Heal.

[15] Bligh EG, Dyer WJ (1959). A rapid method for total lipid extraction and purification. Can J Biochem Physiol.

[16] Folch J, Ascoli I, Lees M, Meath JA, LeBaron FN (1951). Preparation of lipide extracts from brain tissue. J Biol Chem.

[17] Tajima Y, Ishikawa M, Maekawa K (2013). Lipidomic analysis of brain tissues and plasma in a mouse model expressing mutated human amyloid precursor protein/tau for Alzheimer’s disease. Lipids Health Dis.

[18] Milatovic D, Aschner M (2009). Measurement of isoprostanes as markers of oxidative stress in neuronal tissue. Curr Protoc Toxicol.

[19] Breil C, Abert Vian M, Zemb T, Kunz W, Chemat F (2017). “Bligh and Dyer” and Folch methods for solid-liquid-liquid extraction of lipids from microorganisms. comprehension of solvatation mechanisms and towards substitution with alternative solvents. Int J Mol Sci.

[20] Masoodi M, Mir AA, Petasis NA, Serhan CN, Nicolaou A (2008). Simultaneous lipidomic analysis of three families of bioactive lipid mediators leukotrienes, resolvins, protectins and related hydroxy-fatty acids by liquid chromatography/electrospray ionisation tandem mass spectrometry. Rapid Commun Mass Spectrom.

[21] Golovko MY, Murphy EJ (2008). An improved LC-MS/MS procedure for brain prostanoid analysis using brain fixation with head-focused microwave irradiation and liquid-liquid extraction. J Lipid Res.

[22] Gouveia-Figueira S, Nording ML (2015). Validation of a tandem mass spectrometry method using combined extraction of 37 oxylipins and 14 endocannabinoid-related compounds including prostamides from biological matrices. Prostaglandins Other Lipid Mediat.

[23] Yue H, Jansen SA, Strauss KI, Borenstein MR, Barbe MF, Rossi LJ, Murphy E (2007). A liquid chromatography/mass spectrometric method for simultaneous analysis of arachidonic acid and its endogenous eicosanoid metabolites prostaglandins, dihydroxyeicosatrienoic acids, hydroxyeicosatetraenoic acids, and epoxyeicosatrienoic acids in rat brain tissue. J Pharm Biomed Anal.

[24] Wong A, Sagar DR, Ortori CA, Kendall DA, Chapman V, Barrett DA (2014). Simultaneous tissue profiling of eicosanoid and endocannabinoid lipid families in a rat model of osteoarthritis. J Lipid Res.

[25] Ament Z, West JA, Stanley E, Ashmore T, Roberts LD, Wright J, Nicholls AW, Griffin JL (2016). PPAR-pan activation induces hepatic oxidative stress and lipidomic remodelling. Free Radic Biol Med.

[26] Guillemot-Legris O, Masquelier J, Everard A, Cani PD, Alhouayek M, Muccioli GG (2016). High-fat diet feeding differentially affects the development of inflammation in the central nervous system. J Neuroinflammation.

[27] Wang Y, Armando AM, Quehenberger O, Yan C, Dennis EA (2014). Comprehensive ultra-performance liquid chromatographic separation and mass spectrometric analysis of eicosanoid metabolites in human samples. J Chromatogr A.

[28] Arnold C, Markovic M, Blossey K (2010). Arachidonic acid-metabolizing cytochrome P450 enzymes are targets of omega-3 fatty acids. J Biol Chem.

[29] Miller T, Donnelly M, Crago E, Roman D, Sherwood P, Horowitz M, Poloyac S (2009). Rapid, simultaneous quantitation of mono and dioxygenated metabolites of arachidonic acid in human CSF and rat brain. J Chromatogr B.

[30] Leng S, Winter T, Aukema HM (2017). Dietary LA and sex effects on oxylipin profiles in rat kidney, liver, and serum differ from their effects on PUFAs. J Lipid Res.

[31] Chiu C-Y, Smyl C, Dogan I, Rothe M, Weylandt K-H (2017). Quantitative profiling of hydroxy lipid metabolites in mouse organs reveals distinct lipidomic profiles and modifications due to elevated n-3 fatty acid levels. Biology (Basel).

[32] Balvers MGJ, Verhoeckx KCM, Meijerink J, Bijlsma S, Rubingh CM, Wortelboer HM, Witkamp RF (2012). Time-dependent effect of in vivo inflammation on eicosanoid and endocannabinoid levels in plasma, liver, ileum and adipose tissue in C57BL/6 mice fed a fish-oil diet. Int Immunopharmacol.

[33] O’Donnell VB, Maskrey BH, Taylor GW (2009). Lipid signaling protocols.

[34] Maskrey BH, O’Donnell VB (2008). Analysis of eicosanoids and related lipid mediators using mass spectrometry. Biochem Soc Trans.

[35] Harboe M (1959). A method for determination of hemoglobin in plasma by near-ultraviolet spectrophotometry. Scand J Clin Lab Investig.

[36] Kortz L, Dorow J, Becker S, Thiery J, Ceglarek U (2013). Fast liquid chromatography-quadrupole linear ion trap-mass spectrometry analysis of polyunsaturated fatty acids and eicosanoids in human plasma. J Chromatogr B.

[37] Dorow J, Becker S, Kortz L, Thiery J, Hauschildt S, Ceglarek U (2016). Preanalytical investigation of polyunsaturated fatty acids and eicosanoids in human plasma by liquid chromatography–tandem mass spectrometry. Biopreserv Biobank.

[38] Saunders RD, Horrocks LA (1984). Simultaneous extraction chromatography and preparation for high-performance of prostaglandins and phospholipids. Anal Biochem.

[39] Basselin M, Fox MA, Chang L, Bell JM, Greenstein D, Chen M, Murphy DL, Rapoport SI (2009). Imaging elevated brain arachidonic acid signaling in unanesthetized serotonin transporter (5-HTT)-deficient mice. Neuropsychopharmacology.

[40] Hennebelle M, Metherel AH, Kitson AP, Otoki Y, Yang J, Sing K, Lee S, Hammock BD, Bazinet RP, Taha AY (2019). Brain oxylipin concentrations following hypercapnia/ischemia: effects of brain dissection and dissection time. J Lipid Res.

[41] Yue H, Strauss KI, Borenstein MR, Barbe MF, Rossi LJ, Jansen SA (2004). Determination of bioactive eicosanoids in brain tissue by a sensitive reversed-phase liquid chromatographic method with fluorescence detection. J Chromatogr B.

[42] Kohira T, Kita Y, Tokuoka SM, Shiba M, Satake M, Shimizu T (2019). Characterization of supported liquid extraction as a sample pretreatment method for eicosanoids and related metabolites in biological fluids. J Chromatogr B.

[43] Stenson WF (2001). Measurement of prostaglandins and other eicosanoids. Curr Protoc Immunol.

[44] Rodriguez-Navas C, Morselli E, Clegg DJ (2016). Sexually dimorphic brain fatty acid composition in low and high fat diet-fed mice. Mol Metab.

[45] Joffre C, Grégoire S, De Smedt V, Acar N, Bretillon L, Nadjar A, Layé S (2016). Modulation of brain PUFA content in different experimental models of mice. Prostaglandins Leukot Essent Fatty Acids.

[46] Wilson NH (2004) Synthetic eicosanoids. In: Curtis-Prior P (ed) Eicosanoids. Wiley, pp 69–94

[47] Choi S-H, Langenbach R, Bosetti F (2006). Cyclooxygenase-1 and -2 enzymes differentially regulate the brain upstream NF-kappa B pathway and downstream enzymes involved in prostaglandin biosynthesis. J Neurochem.

[48] Kirkby NS, Chan MV, Zaiss AK (2016). Systematic study of constitutive cyclooxygenase-2 expression: role of NF-κB and NFAT transcriptional pathways. Proc Natl Acad Sci U S A.

[49] Ciceri P (2002). Pharmacology of celecoxib in rat brain after kainate administration. J Pharmacol Exp Ther.

