# Supplementary material for: Tissue pretreatment for LC–MS/MS analysis of PUFA and eicosanoid distribution in mouse brain and liver
Source: Anal Bioanal Chem. 2019 Dec 21;412(10):2211–23. doi: 10.1007/s00216-019-02170-w (PMC7118053; doi:10.1007/s00216-019-02170-w)
Supplement: Supplementary file 1 — (PPTX 1.06 mb) [file 216_2019_2170_MOESM1_ESM.pdf]

Analytical and Bioanalytical Chemistry

Electronic Supplementary Material

Tissue pretreatment for LC–MS/MS analysis of PUFAs and eicosanoid distribution in murine brain and liver

Madlen Reinicke, Juliane Dorow, Karoline Bischof, Judith Leyh, Ingo Bechmann, Uta Ceglarek

Additional files available under 10.1007/s00216-019-02170-w

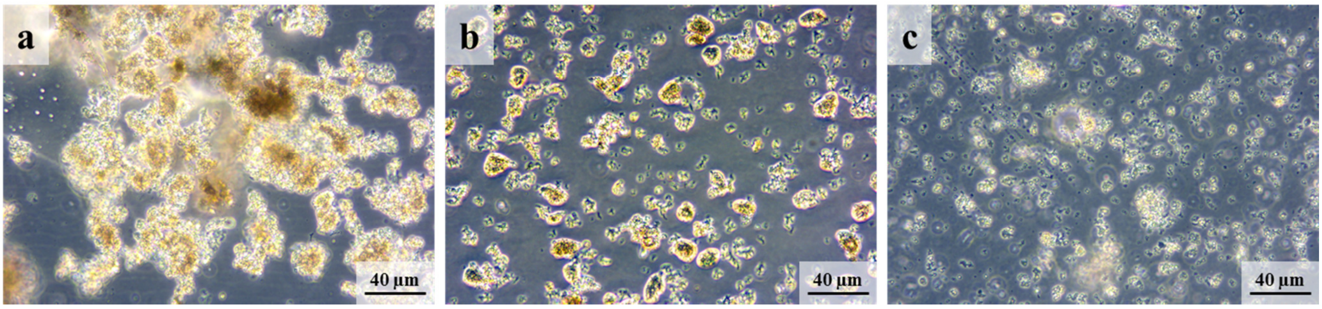

Fig. S1 Microscopic examination of liver homogenates in 20-fold magnification (**a**) Tissue Lyser II®; (**b**) Mikro-Dismembrator S®; (**c**) UCD-300-Bioruptor®

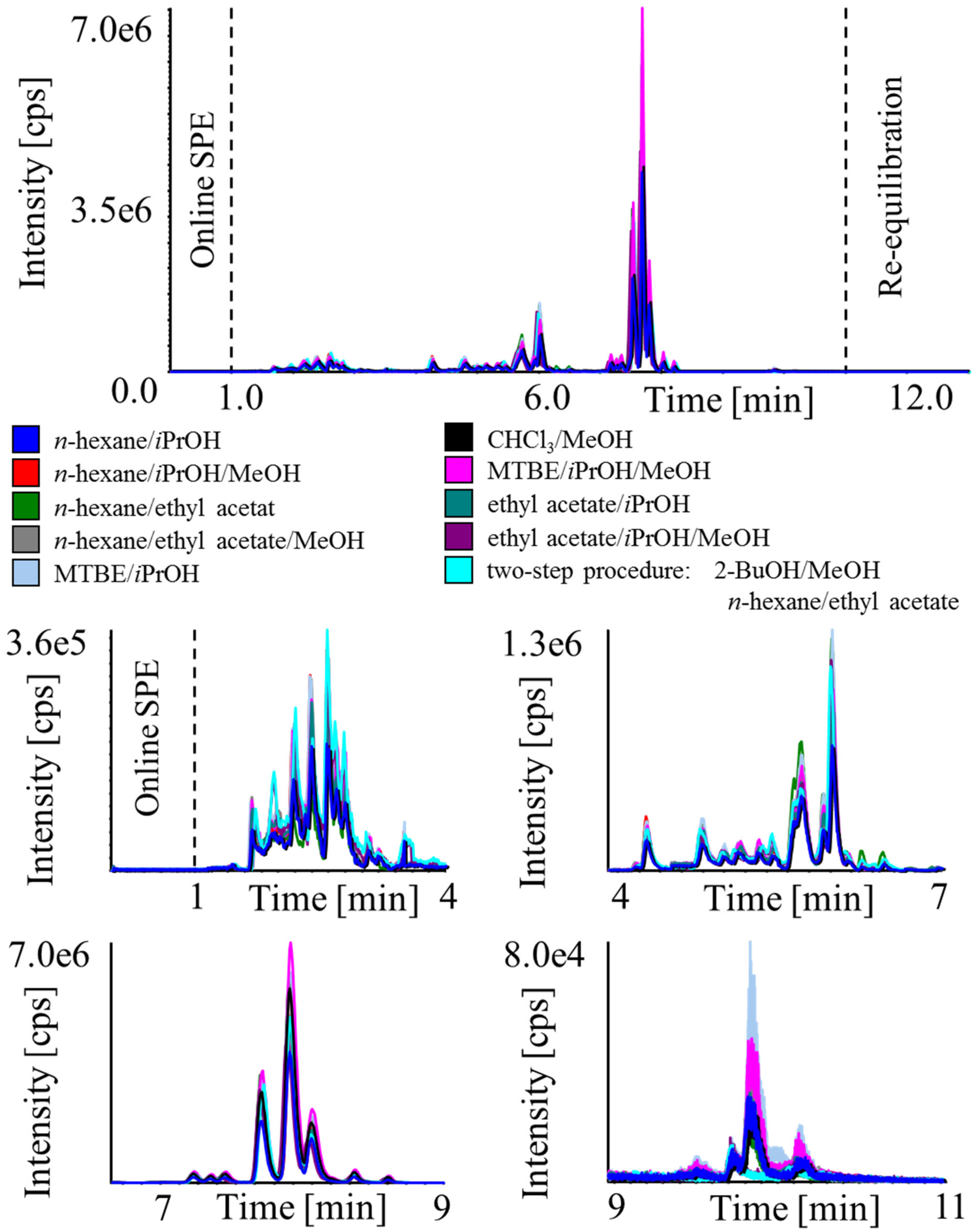

Fig. S2 Chromatogramm (TIC) showed no marked differences between the tested solvent combinations (Table S2)

Table S1 Analyte and internal standard list for PUFAs and eicosanoids (Supplier: Cayman Chemicals, Ann Arbor, Michigan)

Parameter	Common name	Used internal standard	Lipidmaps ID	HMDB
**EPA**	Eicosapentaenoic acid	EPA-d_5	LMFA01030759	HMDB01999
**α-LA**	α-Linolenic acid	EPA-d_5	LMFA01030152	HMDB01388
**γ-LA**	γ-Linolenic acid	EPA-d_5	LMFA01030141	HMDB03073
**DHA**	Docosahexaenoic acid	DHA-d_5	LMFA01030185	HMDB02183
**ARA**	Arachidonic acid	ARA-d_8	LMFA01030001	HMDB01043
**LA**	Linoleic acid	ARA-d_8	LMFA01030120	HMDB00673
**DHGLA**	Dihomo-γ-linolenic acid	ARA-d_8	LMFA01030158	HMDB02925
**TxB_2**	Thromboxane B_2	TxB_2-d_4	LMFA03030002	HMDB03252
**TxB_3**	Thromboxane B_3	TxB_2-d_4	LMFA03030006<br>LMFA03030016	HMDB05099
**11-dehydro-TxB_2**	11-dehydro-Thromboxane B_2	11-dehydro-TxB_2-d_4	LMFA03030014<br>LMFA03030004	HMDB04242
**PGA_2**	Prostglandin A_2	PGE_2-d_4	LMFA03010035	HMDB02752
**PGB_2**	Prostglandin B_2	PGE_2-d_4	LMFA03010018	HMDB04236
**PGD_1**	Prostglandin D_1	PGD_2-d_4	LMFA03010049	HMDB05102
**PGD_2**	Prostglandin D_2	PGD_2-d_4	LMFA03010004	HMDB01403
**PGD_3**	Prostglandin D_3	PGD_2-d_4	LMFA03010142	HMDB03034
**PGE_1**	Prostglandin E_1	PGE_2-d_4	LMFA03010134	HMDB01442
**PGE_2**	Prostglandin E_2	PGE_2-d_4	LMFA03010003	HMDB01220
**PGE_3**	Prostglandin E_3	PGE_2-d_4	LMFA03010135	HMDB02664
**$PGF_{1α}$**	Prostglandin $F_{1α}$	$PGF_{2α}$-d_4	LMFA03010137	HMDB02685

**$PGF_{2\alpha}$**	Prostglandin $F_{2\alpha}$	$PGF_{2\alpha}$-d_4	LMFA03010002	HMDB01139
**$PGF_{3\alpha}$**	Prostglandin $F_{3\alpha}$	$PGF_{2\alpha}$-d_4	LMFA03010138	HMDB02122
**PGJ_2**	Prostglandin J_2	PGE_2-d_4	LMFA03010019	HMDB02710
**11β-PGE_2**	11β-Prostglandin E_2	PGE_2-d_4	LMFA03010060	
**11β-$PGF_{2\alpha}$**	11β-Prostglandin $F_{2\alpha}$	$PGF_{2\alpha}$-d_4	LMFA03010029	HMDB04049
**Tetranor-PGEM**	Tetranor-Prostglandin EM	Tetranor-PGEM-d_6	LMFA03010032	
**Tetranor-PGDM**	Tetranor-Prostglandin DM	Tetranor-PGDM-d_6	LMFA03010221	
**20-hydroxy-PGE_2**	20-hydroxy-Prostglandin E_2	Tetranor-PGEM-d_6	LMFA03010014	HMDB03247
**20-hydroxy-$PGF_{2\alpha}$**	20-hydroxy-Prostglandin $F_{2\alpha}$	Tetranor-PGEM-d_6	LMFA03010029	HMDB04049
**13,14-dihydro-15-keto-tetranor-$PGF_{1\alpha}$**	13,14-dihydro-15-keto-tetranor-Prostglandin $F_{1\alpha}$	$PGF_{2\alpha}$-d_4		
**2,3-dinor-11β-$PGF_{2\alpha}$**	2,3-dinor-11β-Prostglandin $F_{2\alpha}$	8-iso-$PGF_{2\alpha}$-d_4	LMFA03010011	HMDB02277
**6-keto-PGE_1**	6-keto-Prostglandin E_1	PGE_2-d_4	LMFA03010012	HMDB04241
**6-keto-$PGF_{1\alpha}$**	6-keto-Prostglandin $F_{1\alpha}$	$PGF_{2\alpha}$-d_4	LMFA03010001	HMDB02886
**15-keto-PGE_2**	15-keto Prostglandin E_2	PGE_2-d_4	LMFA03010030	HMDB03175
**15-keto-$PGF_{2\alpha}$**	15-keto Prostglandin $F_{2\alpha}$	$PGF_{2\alpha}$-d_4	LMFA03010026	HMDB04240
**8-iso-PGA_2**	8-iso-Prostglandin A_2	PGE_2-d_4		
**8-iso-PGE_2**	8-iso Prostglandin E_2	PGE_2-d_4	LMFA03110003	HMDB05844
**8-iso-$PGF_{2\alpha}$**	8-iso-Prostglandin $F_{2\alpha}$	8-iso-$PGF_{2\alpha}$-d_4		
**8-iso-15-keto-PGE_2**	8-iso-15-keto Prostglandin E_2	PGE_2-d_4	LMFA03110009	HMDB02341
**8-iso-15-keto-$PGF_{2\alpha}$**	8-iso-15-keto-Prostglandin $F_{2\alpha}$	8-iso-$PGF_{2\alpha}$-d_4	LMFA03110005	HMDB05077
**8-iso-13,14-dihydro-15-keto $PGF_{2\alpha}$**	8-iso-13,14-dihydro-15-keto Prostglandin $F_{2\alpha}$	13,14-dihydro-15-keto $PGF_{2\alpha}$-d_4	LMFA03110004	HMDB06562

**11b-13,14-dihydro-15-keto-$PGF_{2α}$**	11b-13,14-dihydro-15-keto-Prostglandin $F_{2α}$	13,14-dihydro-15-keto $PGF_{2α}$-*d_4*	LMFA03010203	
**13,14-dihydro-15-keto-PGE_2**	13,14-dihydro-15-keto-Prostglandin E_2	13,14-dihydro-15-keto $PGF_{2α}$-*d_4*	LMFA03010031	HMDB02776
**13,14-dihydro-15-keto-PGD_2**	13,14-dihydro-15-keto-Prostglandin D_2	13,14-dihydro-15-keto $PGF_{2α}$-*d_4*	LMFA03010022	
**13,14-dihydro-15-keto-$PGF_{2α}$**	13,14-dihydro-15-keto-Prostglandin $F_{2α}$	13,14-dihydro-15-keto $PGF_{2α}$-*d_4*	LMFA03010027	HMDB04685
**15-deoxy-D12,14-PGD_2**	15-deoxy-D12,14-Prostglandin D_2	PGD_2-*d_4*	LMFA03010051	
**15-deoxy-D12,14-PGJ_2**	15-deoxy-D12,14-Prostglandin J_2	PGE_2-*d_4*	LMFA03010021	HMDB05079
**LTB_4**	Leukotriene B_4	LTB_4-*d_5*	LMFA03020001	HMDB01085
**LTC_4**	Leukotriene C_4	LTC_4-*d_5*	LMFA03020003	HMDB01198
**LTD_4**	Leukotriene D_4	LTD_4-*d_5*	LMFA03020077	HMDB03080
**LTE_4**	Leukotriene E_4	LTE_4-*d_5*	LMFA03020075<br>LMFA03020002	HMDB02200
**14,15-LTE_4**	14,15-Leukotriene E_4	LTE_4-*d_5*	LMFA03020033	
**12-oxo-LTB_4**	12-oxo-Leukotriene B_4	LTB_4-*d_4*	LMFA03020024	HMDB04234
**14,15-dehydro-LTB_4**	14,15-dehydro-Leukotriene B_4	LTB_4-*d_4*	LMFA03020017	
**18-carboxy-dinor-LTB_4**	18-carboxy-dinor-Leukotriene B_4	LTB_4-*d_4*	LMFA05000593	
**20-carboxy-LTB_4**	20-carboxy-Leukotriene B_4	LTB_4-*d_4*	LMFA03020016	HMDB06059
**20-hydroxy-LTB_4**	20-hydroxy-Leukotriene B_4	LTB_4-*d_4*	LMFA03020018<br>LMFA03020063	HMDB01509
**5(S),6(R)-LxA_4**	5(S),6(R)-Lipoxin A_4	LTB_4-*d_5*	LMFA03040001	HMDB04385
**5(S),14(R)-LxB_4**	5(S),14(R)-Lipoxin B_4	LTB_4-*d_5*	LMFA03040002	HMDB05082
**5(S)-HETE**	5(S)-hydroxy-eicosatetraenoic acid	5(S)-HETE-*d_8*	LMFA03060002	HMDB11134
**8(S)-HETE**	8(S)-hydroxy-eicosatetraenoic acid	15(S)-HETE-*d_8*		HMDB04679

**9(S)-HETE**	9(S)-hydroxy-eicosatetraenoic acid	15(S)-HETE-d_8	LMFA03060027	HMDB04679
**11(S)-HETE**	11(S)-hydroxy-eicosatetraenoic acid	15(S)-HETE-d_8	LMFA03060003	HMDB04682
**12(S)-HETE**	12(S)-hydroxy-eicosatetraenoic acid	5(S)-HETE-d_8	LMFA03060064<br>LMFA03060007	HMDB06111
**15(S)-HETE**	15(S)-hydroxy-eicosatetraenoic acid	15(S)-HETE-d_8	LMFA03060001	HMDB03876
**16(S)-HETE**	16(S)-hydroxy-eicosatetraenoic acid	15(S)-HETE-d_8		HMDB04680
**17(S)-HETE**	17(S)-hydroxy-eicosatetraenoic acid	15(S)-HETE-d_8	LMFA03060091	
**(±)18-HETE**	(±)18-hydroxy-eicosatetraenoic acid	15(S)-HETE-d_8	LMFA03060092	
**19(S)-HETE**	19(S)-hydroxy-eicosatetraenoic acid	15(S)-HETE-d_8	LMFA03060074	HMDB11136
**20-HETE**	20-hydroxy-eicosatetraenoic acid	20-HETE-d_6	LMFA03060009	HMDB05998
**Tetranor-12(S)-HETE**	Tetranor-12(S)-hydroxy-eicosatetraenoic acid	5(S)-HETE-d_8		
**5(S),6(S)-DiHETE**	5(S),6(S)-dihydroxyeicosatetraenoic acid	15(S)-HETE-d_8	LMFA03060018	
**5(S),15(S)-DiHETE**	5(S),15(S)-dihydroxyeicosatetraenoic acid	15(S)-HETE-d_8	LMFA03060010	HMDB10216
**8(S),15(S)-DiHETE**	8(S),15(S)-dihydroxyeicosatetraenoic acid	15(S)-HETE-d_8	LMFA03060050	HMDB10219
**5(S)-HEPE**	5(S)-hydroxy-eicosapentaenoic acid	15(S)-HETE-d_8	LMFA03070010	HMDB05081
**(±)11-HEPE**	(±)11-hydroxy-eicosapentaenoic acid	15(S)-HETE-d_8	LMFA03070030	
**12(S)-HEPE**	12(S)-hydroxy-eicosapentaenoic acid	15(S)-HETE-d_8	LMFA03070008	HMDB10202
**(±)5,6-DHET**	(±)5,6-dihydroxy-eicosatrienoic acid	(±)8,9-DHET-d_{11}	LMFA03050004	HMDB02343
**(±)8,9-DHET**	(±)8,9-dihydroxy-eicosatrienoic acid	(±)8,9-DHET-d_{11}	LMFA03050006	HMDB02311
**(±)11,12-DHET**	(±)11,12-dihydroxy-eicosatrienoic acid	(±)8,9-DHET-d_{11}	LMFA03050008	HMDB02314
**(±)14,15-DHET**	(±)14,15-dihydroxy-eicosatrienoic acid	(±)8,9-DHET-d_{11}	LMFA03050010	HMDB02265
**12(S)-HHT**	12(S)-hydroxy-heptadecatrienoic acid	15(S)-HETE-d_8	LMFA03050002	HMDB0012535

**(±)9-HODE**	(±)9-hydroxy-octadecadienoic acid	13(S)-HODE-d_4	LMFA02000151<br>LMFA02000036<br>LMFA02000188	HMDB04670
**(±)13-HODE**	(±)13-hydroxy-octadecadienoic acid	13(S)-HODE-d_4	LMFA02000190<br>LMFA02000035<br>LMFA02000228	HMDB06939
**5-oxo-ETE**	5-oxo-eicosatetraenoic acid	5-oxo-ETE-d_7	LMFA03060011	HMDB10217
**12-oxo-ETE**	12-oxo-eicosatetraenoic acid	5-oxo-ETE-d_7	LMFA03060019	
**15-oxo-ETE**	15-oxo-eicosatetraenoic acid	5-oxo-ETE-d_7	LMFA03060051	HMDB10210
**5(S)-HpETE**	5(S)-hydroperoxyeicosatetraenoic acid	5(S)-HETE-d_8	LMFA03060012	HMDB11135
**12(S)-HpETE**	12(S)-hydroperoxyeicosatetraenoic acid	5(S)-HETE-d_8	LMFA03060013	HMDB04692
**15(S)-HpETE**	15(S)-hydroperoxyeicosatetraenoic acid	5(S)-HETE-d_8	LMFA03060014	HMDB04244
**(±)5,6-EET**	(±)5,6-epoxyeicosatrienoic acid	(±)8,9-DHET-d_{11}	LMFA03080002	HMDB02190
**(±)8,9-EET**	(±)8,9-epoxyeicosatrienoic acid	(±)8,9-DHET-d_{11}	LMFA03080003	HMDB02232
**(±)11,12-EET**	(±)11,12-epoxyeicosatrienoic acid	(±)8,9-DHET-d_{11}	LMFA03080004	HMDB10409
**(±)14,15-EET**	(±)14,15-epoxyeicosatrienoic acid	(±)8,9-DHET-d_{11}	LMFA03080005	HMDB04264
**Tetranor-PGEM-d_6**	Tetranor-Prostglandin EM-d_6			
**Tetranor-PGDM-d_6**	Tetranor-Prostglandin DM-d_6			
**8-iso-PGF$_{2\alpha}$-d_4**	8-iso-Prostglandin F$_{2\alpha}$-d_4			
**TxB$_2$-d_4**	Thromboxane B$_2$-d_4			
**PGF$_{2\alpha}$-d_4**	Prostglandin F$_{2\alpha}$-d_4			
**PGE$_2$-d_4**	Prostglandin E$_2$-d_4			
**11-dehydro-TxB$_2$-d_4**	11-dehydro-Thromboxane B$_2$-d_4			
**PGD$_2$-d_4**	Prostglandin D$_2$-d_4			

**LTD_4-d_5**	Leukotriene D_4-d_5
**13,14-dihydro-15-keto-$PGF_{2\alpha}$-d_4**	13,14-dihydro-15-keto-Prostglandin $F_{2\alpha}$-d_4
**LTC_4-d_5**	Leukotriene C_4-d_5
**LTE_4-d_5**	Leukotriene E_4-d_5
**LTB_4-d_4**	Leukotriene B_4-d_5
**(±)8,9-DHET-d_{11}**	(±)8,9-Dihydroxy-eicosatrienoic acid-d_{11}
**13(S)-HODE-d_4**	13(S)-Hydroxy-octadecadienoic acid-d_4
**5(S)-HETE-d_8**	5(S)-Hydroxy-eicosatetraenoic acid-d_8
**15(S)-HETE-d_8**	15(S)-Hydroxy-eicosatetraenoic acid-d_8
**20-HETE-d_6**	20-Hydroxy-eicosatetraenoic acid-d_6
**5-oxo-ETE-d_7**	5-oxo-Eicosatetraenoic acid-d_7

Tables S2, S3 and **S5 to S8** see separate Excel files.

Table S4 Relative changes of detected PUFAs and eicosanoids in liver after 3 and 30 days of storage at
-80 °C. Sample aliquots were either dried or contained extraction solvent (*n*-hexane/*i*PrOH (60:40 v/v). Dried condition and direct analysis without storage was set as 100 %

metabolite	dried, 3 days stored	dried, 30 days stored	in solvent, 3 days stored	in solvent, 30 days stored
	% Change ± SD	% Change ± SD	% Change ± SD	% Change ± SD
EPA	101 ± 5	102 ± 9	130 ± 16	170 ± 32
α-/γ-LA	101 ± 6	102 ± 10	128 ± 15	155 ± 29
DHA	101 ± 4	98 ± 4	126 ± 17	154 ± 28
ARA	100 ± 3	100 ± 3	120 ± 11	153 ± 27
LA	101 ± 3	107 ± 3	120 ± 15	174 ± 31
DHGLA	100 ± 3	112 ± 2	124 ± 17	184 ± 29
Tetranor-PGEM/ Tetranor-PGDM	109 ± 4	116 ± 4	120 ± 1	131 ± 15
6-keto-$PGF_{1α}$	95 ± 15	94 ± 5	100 ± 17	84 ± 5
8-iso-$PGF_{2α}$	102 ± 14	95 ± 10	111 ± 29	92 ± 10
11β-$PGF_{2α}$	100 ± 11	102 ± 9	107 ± 21	92 ± 4
PGE_3	103 ± 16	133 ± 1	138 ± 15	204 ± 31
$PGF_{2α}$	103 ± 14	101 ± 13	114 ± 23	95 ± 1
PGD_3	103 ± 19	169 ± 23	141 ± 5	217 ± 31
5,6-DHET	108 ± 12	102 ± 8	112 ± 14	108 ± 12
8,9-DHET	100 ± 5	96 ± 8	116 ± 14	117 ± 17
11,12-DHET	100 ± 8	98 ± 8	119 ± 17	124 ± 18
14,15-DHET	111 ± 10	105 ± 8	126 ± 17	133 ± 18
12-HHT	105 ± 9	104 ± 6	128 ± 18	118 ± 14
11-HEPE	96 ± 12	94 ± 5	110 ± 14	100 ± 12
12-HEPE	98 ± 13	92 ± 7	110 ± 16	101 ± 12
8-HETE	95 ± 13	100 ± 1	107 ± 11	98 ± 1
9-HETE	99 ± 15	94 ± 3	114 ± 15	97 ± 6
11-HETE	99 ± 11	101 ± 4	110 ± 9	101 ± 4
12-HETE	101 ± 15	107 ± 2	116 ± 11	105 ± 2
15-HETE	109 ± 9	112 ± 4	121 ± 16	125 ± 10
16-HETE	99 ± 8	100 ± 9	125 ± 16	113 ± 15
17-HETE	104 ± 13	99 ± 12	142 ± 16	142 ± 16
18-HETE	102 ± 11	99 ± 8	127 ± 21	122 ± 24
9-HODE	96 ± 12	91 ± 6	108 ± 15	106 ± 13
13-HODE	100 ± 13	95 ± 7	113 ± 13	109 ± 10
15-oxo-ETE	107 ± 18	169 ± 4	101 ± 17	193 ± 41
